# Is there any relationship between red blood cell distribution width and prognosis of brain death?

**DOI:** 10.22088/cjim.14.1.37

**Published:** 2023

**Authors:** Sanaz Dehghani, Elahe Pourhosein, Amir Ali Hamidieh, Zeinab Mansouri, Niloufar Tirgar, Fariba Namdar, Pantea Ramezannezhad, Arefeh Jafarian, Marzieh Latifi

**Affiliations:** 1 Organ Procurement Unit, Sina Hospital, Tehran University of Medical Sciences, Tehran, Iran; 2 Iranian Tissue Bank & Research Center, Tehran University of Medical Sciences, Tehran, Iran; 3 Pediatric Cell Therapy Research Center, Tehran University of Medical Sciences, Tehran, Iran; 4 Department of Emergency Medicine, School of Medicine, Kashani Hospital, Shahrekord University of Medical Sciences, Shahrekord, Iran; € Arefeh Jafarian and Marzieh Latifi contributed equally in this manuscript

**Keywords:** RDW, Brain death, Cardiac arrest

## Abstract

**Background:**

Accumulating evidence has demonstrated that RDW (red blood cell distribution width) may independently predict clinically important outcomes in many populations. However, the role of RDW has not been elucidated in brain death. We conducted this study with the aim of evaluating the predictive value of RDW in brain death.

**Methods:**

A retrospective study of seventy-seven of brain death cases during 36 months were evaluated at university hospitals, affiliated in Tehran, Iran. Demographical data include age, sex, BMI and cause of brain death, also laboratory results (red blood cell distribution, mean corpuscular volume, hemoglobin) collected by checklists from patient records. Having the three RDW measurements (days of hospital admission, day of brain death, and day of cardiac arrest) required.

**Results:**

Time interval from hospital admission until brain death was 5.27±4.07. The mean age of brain death cases was 32.65±16.53. The mean RDW values on days of hospital admission, the day of brain death, and the day of cardiac arrest were 14.53±1.98, 15.12±1.93 and 15.18±2.07, respectively. Results of the repeated-measures ANOVA test reveal that RDW level was constantly higher in the traumatic patient group compared to the non-traumatic ones (P=0.008).

**Conclusion:**

The frequency of brain death was high in patients with high RDW values. RDW might be a prognostic biomarker for brain death. More prospective studies with large sample size and long follow-up period should be carried out to determine the prognostic significance of RDW and brain death in future.

Brain death is characterized by the irreversible loss of brain, including the brain stem and cortex function (1, 2). ICU staff plays a crucial role in the management of brain death cases through identifying potential donors, declaration of brain death, and providing appropriate medical care (3). Certain criteria need to be fulfilled for diagnosis of brain death such as irreversible coma, absence of brain stem reflexes, and lack of self-respiration (4-7). Biomarkers and neurological tests can help physicians avoid futile care by predicting poor outcomes early after ICU admission (8). Detection of new prognostic markers may identify at-risk patients early enough. One of these markers is red cell distribution width (RDW). RDW test measures the amount of red blood cell variation in volume and size, reported in routine blood tests (9).Given that the RDW is routinely reported by clinical laboratories as a component of the complete blood count (CBC), understanding its prognosis could be very valuable for risk stratification in clinical decision making (10). 

RDW evidently increases in various pathological conditions, including heart diseases (11-13) and ischemic cerebrovascular disease (14), inflammatory bowel disease, pulmonary disease (PD), cerebrovascular diseases (15), as well as in hypertensive patients (4, 13, 16, 17). Increased RDW is a symptom of disruption and problem in the red blood cell production process due to metabolic and/or biological imbalances. Metabolic and biological disorders include telomere shortening, oxidative stress, inflammation, malnutrition, impaired fat metabolism, hormonal imbalance, increased blood pressure and decreased tissue repair potential (18). As few studies have been carried out in this regard, more evidence and studies are needed. Therefore, the current study inspired us to explore whether RDW could be used as a prognostic biomarker for brain death.

## Methods

This is a retrospective study for the purpose of examining the relationship between RDW and prognosis of brain death. All brain death cases in 60 hospitals affiliated with Sina and Emam organ procurement units (OPU’s) in Iran during 2017-2020 (36 months) were considered as a sample. A study protocol was approved by the Ethics Committee of Tehran University of Medical Sciences (TUMS). (ID: IR.TUMS.IKHC.REC.1400.020). 

The full clinical record of the cases was registered at inclusion with the detailed routine laboratory tests. Cases were excluded with any illness possibly affecting RDW levels (such as thalassemia trait, hereditary helliptocytosis, hemoglobin C disease, hypertension, diabetes, etc.).

Demographic data, history of drug abuse, medicine, as well as clinical and laboratory data were recorded according to research made checklist. By monitoring their clinical and laboratory data, their cardiac arrest time was also recorded. The information was then reviewed by two ICU nurses for deleting wrong or dubious records. Recorded laboratory parameters included baseline complete blood count (CBC), RDW, which was performed on whole blood samples collected from brain death cases, whose time of admission, brain death, and cardiac arrest were recorded. Received blood transfusion before hematological testing or had anemia nor thalassemia, hepatitis C and B antigen positive, heart diseases, ischemic cerebrovascular disease, inflammatory bowel disease, pulmonary disease, cerebrovascular diseases, hypertension and all cases who had intake any drugs which can have effect on RDW were excluded to this study. Of the 597 recorded cases successfully checked by two trained researchers, only eighty-five cases included the three stages of RDW measurement. Eight of brain death cases had some exclusion criteria. Finally, seventy-seven cases had inclusion criteria for this study. Laboratory measurements Hemoglobin 1,2,3 and the RDW 1,2,3 were determined in two main OPU’s administration medical centers with the use of a CBC analyzer. The analysis was performed within 2 h after blood collection using an automated cell counter (Sysmex Poch-100iV Diff) which provided the following parameters: total red blood cells (RBC), hemoglobin (Hb), hematocrit (HT), mean corpuscular volume (MCV), mean corpuscular and standard deviation in red cell distribution width (RDW-SD). RDW is a continuous parameter, and its reference average is 14.5% in men and 11.5% in women (19).

**Statistical methods: **Summary statistics for the continuous variables were presented as mean ± SD, and as numbers and percentages for categorical variables. The mean changes in continuous outcome variables among three groups were assessed with repeated measure analysis of variance. A p-value less than 0.05 was considered statistically significant. All data were analyzed using SPSS16 software.

## Results

A total of 597 patients were identified during 2017- 2020, seventy-seven of which were included in the study, having the three RDW measurements required. Of those, 31 (40.3%) were women and 46 (59.7%) were men. Time interval from hospital admission until brain death was 5.27±4.07 (median: 4 days). The main characteristics of the study samples are shown in table 1. 

**Figure 1:**
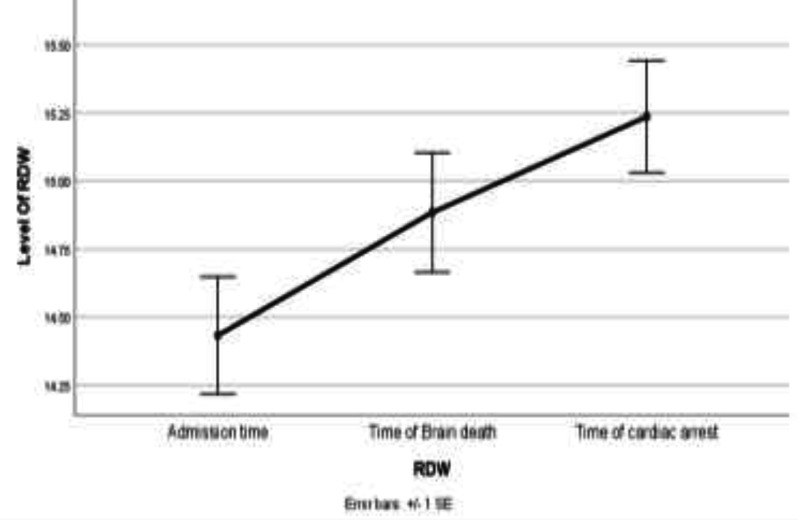
Line chart of red blood cell distribution width (RDW) values in admission time, time of brain death and time of cardiac arrest

The mean RDW values on days of hospital admission, day of brain death, and day of cardiac arrest were 14.53±1.98, 15.12±1.93 and 15.18±2.07, respectively. RDW cases ‘values at the time of brain death are higher than RDW values at the time of admission. RDW levels were constantly increasing throughout the study and there was a significant difference between each time point of RDW measurement. RDW level at the time of brain death was 0.45-fold higher (P=0.002) compared to the time of admission, and additionally 0.8-fold higher (P=0.002) at the time of cardiac arrest compared to time of brain death. The standardized residuals at the-three-time points showed an approximate normal distribution in figure 1. In addition, hemoglobin levels did not change significantly from the time of admission to the time of brain death and significantly decreased after brain death. There was a difference between each time point of hemoglobin measurement. The 0.46-fold change between time of admission and time of brain death was not statistically significant (P=0.093). 

**Table 1:** Baseline characteristics of study population

Variables	
Age (Mean ±SD)	32.65±16.53
BMI (Mean ±SD)	24.83±4.29
GCS (admission time, median)	3
ALT1 (Mean ±SD)	74.4±81
ALT2 (Mean ±SD)	90.9 ±107
ALT3 (Mean ±SD)	88.5±16.53
AST1 (Mean ±SD)	124.8±137.1
AST2 (Mean ±SD)	121.8±134
AST3 (Mean ±SD)	111.7±133.2
Sex, No (%)	
Female	31 (40.3)
Male	46 (57.9)
Cause of brain death, No (%)	
Head trauma ICH-IVH Toxicity Ischemic CVA Post-CPR Tumor Others	28 (36.4) 25 (32.5) 3 (3.9) 6 (7.8) 1 (1.3) 3 (3.9) 11 (14.3)
Blood group, No (%)	
O A B AB	23 (29.9) 28 (36.4) 22 (28.6) 4 (5.2)
Smoker, No (%)	
Yes	20 (26)
No	57 (74)
Addiction, No (%)	
Yes	5 (6.5)
No	72 (93.5)
CPR, No (%)	
Yes	14 (20.3)
No	55 (79.7)
Shock	
Yes	3 (3.9)
No	16 (84.2)

**Figure 2:**
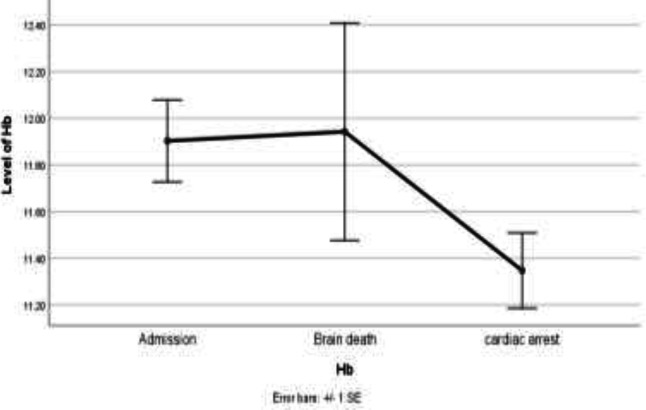
Line chart of Hemoglobin (Hb) in admission time, brain death time and cardiac arrest time

**Figure 3:**
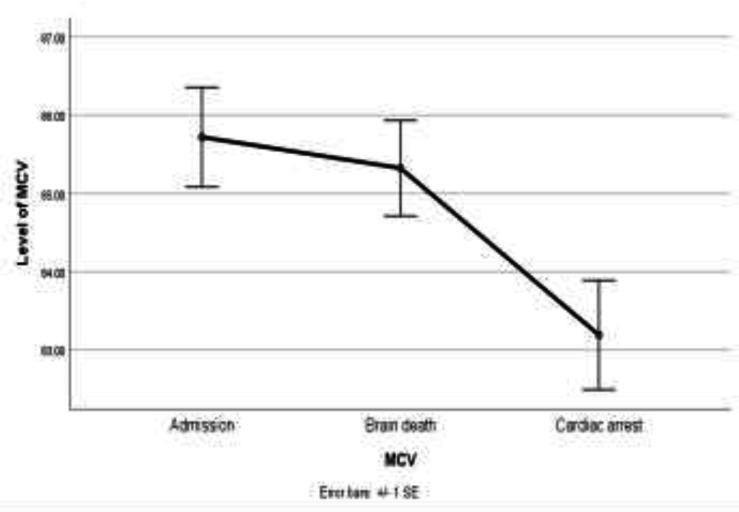
Line chart of MCV in admission time, brain death time and cardiac arrest time

However, the 0.55-fold change between the time of cardiac arrest compared to the time of admission was statistically significant (P=0.002) (Figure 2). MCV did not significantly change from the time of admission to time of brain death, though significantly decreased after brain death. It was decreased by 0.39-fold (P=0.52) at the time of brain death compared to the time of admission, but by 2.52-fold (P=0.001) at the time of cardiac arrest compared to the time of admission. (Figure 3).

**Figure 4:**
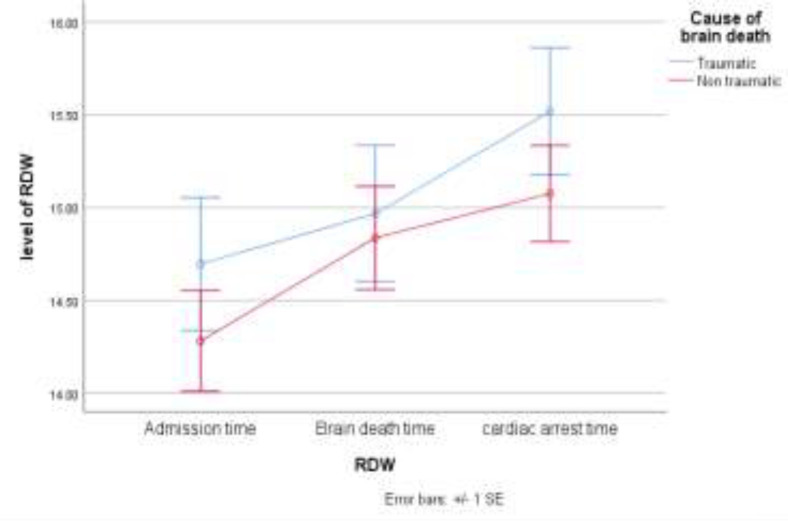
Line chart comparison of red blood cell distribution width (RDW) and causes of brain death

Results of the repeated-measures ANOVA test reveal that RDW level was constantly higher in the traumatic patient group compared to the non-traumatic ones, being 0.32-fold higher (P=0.008) (Figure 4). 

On the contrary, there was not a significant difference between the level of RDW in three stages (time of admission, time of brain death, time of cardiac arrest) with BMI (P=0.92), smoking (P=0.85), and addiction (P=0.91). In univariate correlation analysis, there was a negative correlation between RDW_1 _with Hb_1_ (r=-0.397; P=0.000), Hb_2_ (r=-0.298; p=0.012), Hb_3_ (r=-0.265; P=0.017), MCV_1_ (r=-0.401; P=0.000), MCV_2_ (r=-0.686; P= 0.000), MCV_3_ (r=-0.498; P= 0.0100). 

Further information about the correlation between admission RDW_2,3_ level and other variables are shown in table 2.

**Table 2:** Univariate correlation analysis between Admission RDW Levels and Blood Factors in the Study Sample

	**Variables**	**r**	**P value**
RDW1	Hb1	-0.397	0.000*
	Hb2	-0.298	0.012*
	Hb3	-0.265	0.017*
	MCV1	-0.401	0.000*
	MCV2	-0.686	0.000*
	MCV3	-0.498	0.000*
	RDW2	0.831	0.000*
	RDW3	0.697	0.000*
RDW2	Hb1	-0.331	0.001*
	Hb2	-0.286	0.003*
	Hb3	-0.249	0.009*
	MCV1	-0.410	0.000*
	MCV2	-0.474	0.000*
	MCV3	-0.292	0.000*
RDW3	Hb1	-0.248	0.000*
	Hb2	-0.036	0.571
	Hb3	-0.045	0.437
	MCV1	-0.377	0.000*
	MCV2	-0.436	0.000*
	MCV3	-0.294	0.000*

AST1: Hb1: Hb level at the time of admission; Hb2: Hb level at the time of brain death; Hb3: Hb level at the time of cardiac arrest; MCV1: MCV level at the time of admission; MCV2: MCV level at the time of brain death; MCV3: MCV level at the time of cardiac arrest; RDW1: RDW level at the time of admission; RDW2: RDW level at the time of brain death; RDW3: RDW in the time of cardiac arrest.

## Discussion

Intriguing evidence has recently revealed that the RDW may provide valuable information for prognosis of a variety of disorders such as cardiovascular diseases (20), cancer (21), chronic lung diseases (17), and acute stroke (22), as well as for planning the short- and long-term prognosis in patients with these pathological conditions (23, 24). Taken together, the results of our study attest that RDW values at the time of admission was a prognostic value of changes in RDW levels during length of hospitalization. According to our results, RDW values at the time of brain death are higher than RDW values at the time of admission (P =0.001). Furthermore, the RDW level on the day of cardiac arrest was also significantly higher than on the day of brain death diagnosis (P =0.001). Only one previous study by Nevzat Mehmet looked at RDW’s prediction of brain death (25). They reveal that the RDW levels on the days of brain death and cardiac arrest were significantly higher than on the day of admission (P =0.001). Moreover, the RDW level on the day of cardiac arrest was significantly higher than on the day of brain death (P =0.001). Their results are in line with ours. Therefore, RDW could play a role in prediction of neurologic outcome in brain death cases. The result of Nevzat and et al. confirm our result that, RDW could be used as a supportive diagnostic biomarker for diagnosis of brain death and RDW is a useful biomarker to support clinical diagnosis (21). Another study by Biao Zhang(26) compared RDW level between survival and non-survival head trauma groups. They showed that RDW is a predictor of mortality in patients with TBI. According to our result, there was a significant difference between the level of RDW_1, 2_, _3_ and cause of brain death and RDW was significantly higher in subgroups of traumatic patients. Giuseppe et al. (27) in 2016 showed that the RDW values increased in trauma patients, especially in those with head trauma. Another study by Biao Zhang (26) in 2015 revealed that RDW is a predictor of mortality in patients with traumatic brain injury. Lee et al. similar to our results, revealed that RDW can independently predict mortality in trauma patients (28). 

In conclusion, RDW might be a prognostic biomarker for brain death. In patients with loss of consciousness due to brain injury, a high RDW might be associated with a higher risk of brain death. Clinicians should pay attention to the RDW level. In summary, we have shown that the admission of RDW level might be a powerful independent prognostic factor for predicting brain death. Considering the fact that no similar research data could be referred to, there were a number of limitations in the present study, the most important of which was missing a large number of RDW_1_ measurements. On the contrary, our study was performed in two main OPUs with 60 affiliated hospitals. The strength of our study was the distribution of these hospitals throughout the country. Therefore, our results may be applicable to other institutions with different patient populations and larger sample size with a wider variety of demographic characteristics.
